# FPGA-based Fused Smart Sensor for Real-Time Plant-Transpiration Dynamic Estimation

**DOI:** 10.3390/s100908316

**Published:** 2010-09-02

**Authors:** Jesus Roberto Millan-Almaraz, Rene de Jesus Romero-Troncoso, Ramon Gerardo Guevara-Gonzalez, Luis Miguel Contreras-Medina, Roberto Valentin Carrillo-Serrano, Roque Alfredo Osornio-Rios, Carlos Duarte-Galvan, Miguel Angel Rios-Alcaraz, Irineo Torres-Pacheco

**Affiliations:** 1 CA Ingenieria de Biosistemas, Division de Investigacion y Posgrado, Facultad de Ingenieria, Universidad Autonoma de Queretaro, Cerro de las Campanas s/n, 76010, Queretaro, Qro., Mexico; E-Mails: roberto.millan@uaq.mx (J.R.M.-A.); ramon.guevara@uaq.mx (R.G.G.-G.); mcontreras@hspdigital.org (L.M.C.-M.); cduarte20@alumnos.uaq.mx (C.D.-G.); mrios24@alumnos.uaq.mx (M.A.R.-A.); 2 HSPdigital-CA Telematica, DICIS, Universidad de Guanajuato, Carr. Salamanca-Valle km 3.5+1.8, Palo Blanco, 36885 Salamanca, Gto., Mexico; E-Mail: troncoso@hspdigital.org (R.J.R.-T.); 3 HSPdigital-CA Mecatronica, Facultad de Ingenieria, Universidad Autonoma de Queretaro, Campus San Juan del Rio, Rio Moctezuma 249, 76807 San Juan del Rio, Qro., Mexico; E-Mail: raosornio@hspdigital.org (R.A.O.-R.); 4 Division de Investigacion y Posgrado, Facultad de Ingenieria, Universidad Autonoma de Queretaro, Cerro de las Campanas s/n, 76010, Queretaro, Qro., Mexico; E-Mail: roberto.carrillo@uaq.mx (R.V.C.-S.)

**Keywords:** smart sensor, transpiration, stomatal conductance, precision agriculture, phytomonitoring, water stress, field programmable gate array

## Abstract

Plant transpiration is considered one of the most important physiological functions because it constitutes the plants evolving adaptation to exchange moisture with a dry atmosphere which can dehydrate or eventually kill the plant. Due to the importance of transpiration, accurate measurement methods are required; therefore, a smart sensor that fuses five primary sensors is proposed which can measure air temperature, leaf temperature, air relative humidity, plant out relative humidity and ambient light. A field programmable gate array based unit is used to perform signal processing algorithms as average decimation and infinite impulse response filters to the primary sensor readings in order to reduce the signal noise and improve its quality. Once the primary sensor readings are filtered, transpiration dynamics such as: transpiration, stomatal conductance, leaf-air-temperature-difference and vapor pressure deficit are calculated in real time by the smart sensor. This permits the user to observe different primary and calculated measurements at the same time and the relationship between these which is very useful in precision agriculture in the detection of abnormal conditions. Finally, transpiration related stress conditions can be detected in real time because of the use of online processing and embedded communications capabilities.

## 1. Introduction

Plant transpiration is the process in which plants exchange moisture with the atmosphere [1]. This process is carried out when plants perform photosynthesis. While the plants are absorbing the carbon dioxide (CO_2_) they also lose a certain amount of water and release oxygen O_2_ [2]. Also, transpiration is performed to maintain temperature equilibrium between plants and their environments, dissipating undesirable heat in the lost water vapor. Plant monitoring commonly includes the estimation of photosynthesis itself, the assimilation or CO_2_ uptake and water thermodynamic relations such as: transpiration (*E*), stomatal conductance (*C_leaf_*), vapor pressure deficit (*VPD*), and leaf-air temperature difference (*LATD*) [3]. Those variables constitute transpiration dynamic indicators which are often used in agriculture to optimize the available water resources [4].

*E* is considered one of the most important plant physiological functions because it encompasses plants evolving and adapting to exchange moisture in a very dry atmosphere that can dehydrate or eventually kill the plant [1]. *C_leaf_* is a transpiration variable that represents a quantitative measurement of the stomatal resistance (*r_s_*) inverse of plant guard cells plus the inverse boundary resistance (*r_b_*) against water vapor flux. Those guard cells act as flux valves to control the water vapor movement from plant to the atmosphere and CO_2_ movement in an inverse way [5]. *LATD* is the difference between air temperature (*T_a_)* and leaf temperature (*T_leaf_*) in relation to the global transpiration process which is proportional to *E*. Furthermore, *VPD* is also a response variable that is calculated by subtracting air vapor pressure (*e_i_*) content from saturation vapor pressure (*e_s_*). These variables are very important because they can indicate drought stress conditions and condensation problems that may cause dangerous plant diseases [2,6,7].

Because of this, transpiration dynamic measurement is crucial and necessary to establish comparisons and understand plant-soil-atmosphere relationships at leaf, plant, canopy, or community levels as well as their interaction and response to environmental [6], chemical [8], or biological [9] factors that generate different stress conditions. Therefore, continuous monitoring of the aforementioned transpiration dynamics by a single smart sensor system is highly desirable. As a consequence, more accurate measurement methods are required to gather more knowledge about these processes. Relative humidity (*RH*) capacitive sensors and thermistors are the most commonly utilized sensors to measure these variables in environmental and agricultural research [2,6,10,11]; however, in modern instrumentation the use of intelligent sensors with *in situ* signal processing capabilities to calculate response variables equations from simple sensor measurements is necessary [12–14]. *E* and *C_leaf_* calculation is based mainly on water vapor exchange measurements [2,6]. This method consists of temporally isolating a plant leaf sample in a miniature gas exchange chamber which is often used for photosynthesis measurements [15]. An air flow is introduced into the leaf chamber to measure the intake *e_i_* and the amount of leaf out vapor (*e_o_*). The absolute amount of water is calculated using *RH* sensors and vapor curve equations from Mollier diagrams by expressing, *E* and *C_leaf_* as vapor mass for each surface unit of each time unit [6,16,17]. Previous monitoring systems have used this technique to obtain *E* and *C_leaf_* from air relative humidity (*RH_a_*) and *T_a_* [18,19]. Temperature, light, carbon, and *RH* measurements contain merged transpiration and photosynthesis dynamics information; therefore, the extraction of those response variables is desirable for precision agriculture applications. Previously, *T_a_* and *RH* sensors have been used in data acquisition systems for environmental monitoring and greenhouse climate controllers [2]. More advanced applications involve offline crop water stress detection based on *E* behavior analysis [20]. Forestry research has also used transpiration dynamics to investigate the properties of trees [21]. Intelligent irrigation has been investigated in order to schedule irrigation cycles according to the speaking plant concept approach, better known as phytomonitoring technique [20,22]. It takes into consideration the plant as the final user of the irrigation line, activating water delivery when plant has an excessive *E. VPD* has been studied in greenhouse climate controller design also in order to determine when *RH* is near to dew point to avoid excessive fogging and consequently leaf condensation that leads to plant diseases [7]. However, those systems do not fuse their sensors data with other transpiration-related response variables such as ambient light and *LATD* nor do they have online *in situ* signal processing capabilities to make real-time decisions. Consequently, it involves having an agricultural expert technician to manually download data to be analyzed offline with at least a one day delay [22]. In precision agriculture, a one day delay can sometimes represent the loss of the total crop. It makes necessary the development of a real time transpiration dynamics intelligent sensor to early detect stress and disorder conditions.

The contribution of this project is to develop a smart sensor capable of estimating plant transpiration dynamic variables: *E*, *C_leaf_*, *LATD*, and *VPD*, through the fusion of five primary low-cost sensors: two *RH* capacitive sensors, two Resistance Temperature Detector (RTD) sensors, one light quantum sensor, average atmospheric pressure data, and fixed volumetric air flow. All the aforementioned instrumentation was embedded into a smart sensor system using an aluminum/acrylic leaf chamber with automatic open/close mechanism based on a miniature servomotor to perform temporal leaf isolation cycles. A vacuum pump is used to generate the air flow through the leaf chamber. Transpiration dynamic response variables are extracted from the primary sensors and its computation is performed *in situ* using digital signal processing techniques such as: average decimation filters, infinite-impulse-response (IIR) filters, polynomial fitting, and the corresponding *E*, *C_leaf_*, *LATD*, and *VPD* equations. The light sensor is fused as a reference to understand daylight information which is related to the beginning of daily transpiration dynamic processes. The data acquisition systems, aforementioned computations, data communication and leaf chamber servomotor/vacuum pump control system are implemented in a field programmable gate array (FPGA) as an embedded smart sensor approach.

## 2. Background

### 2.1. Plant Transpiration Water-Atmosphere Scheme

**Figure 1.**
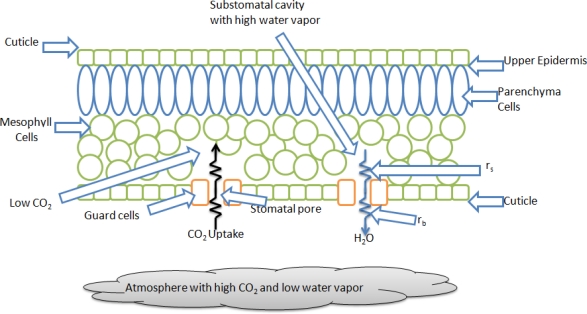
Leaf cut water scheme, showing CO_2_ and H_2_O flows.

In Figure 1, a plant leaf cut scheme is shown where it can be noticed that the different plant tissues (parenchyma, mesophyll and guard cells) which have low CO_2_ contents and a high amount of water. The atmosphere, presented as a gray cloud constitutes a relatively dry environment that can eventually dehydrate or even kill the entire plant if the environmental conditions are not adequate [1]. The stomata guard cells which are the orange ones in Figure 1, are the plant system that controls the stomatal pores open and close process to balance the CO_2_ and water fluxes between the plant and its environment. Here *r_b_* is the boundary resistance and *r_s_* is the stomatal resistance.

### 2.2. Transpiration Process

As was aforementioned, *E* is a function that depends primarily on the difference between *e_i_* and *e_o_*. However, primary humidity sensors provide relative humidity measurement values [6] and need to be converted into *e_i_* and *e_o._* First, it is necessary to determine *e_s_* in order to know the maximum amount of water that air can contain at a specified *T_a_* by using vapor curves in the Mollier thermodynamic diagrams [2] or by using the simplified equation (1) as was previously reported [19]. Then *e_i_* and *e_o_* can easily be obtained with (2) and (3), where *RH_i_* is air input *RH* and *RH_o_* is leaf chamber output *RH*.

(1) $$e_{s}=6.13753\times 10^{-3}\text{exp}\left(T_{a}\frac{18.564-\frac{T_{a}}{254.4}}{T_{a}+255.57}\right)$$

(2) $$e_{i}=\frac{\left({\mathrm{RH}}_{i})(e_{s}\right)}{100}$$

(3) $$e_{o}=\frac{\left({\mathrm{RH}}_{o})(e_{s}\right)}{100}$$

In order to estimate *E*; it is necessary to calculate another important factor, *W* which is the mass flow rate per leaf area, expressed in mol/m^2^/s for open flow systems. *W* equation is stated in (4), where *P* is the atmospheric pressure in Bar, *V* is the volumetric air flow in liters per minute (lpm), *T_a_K* is air temperature in Kelvin (K) and *A* is leaf area in cm^2^, which is often used the effective leaf chamber area in transpiration and photosynthesis measurement systems [19]. The 2005.39 constant is an adjusted coefficient to change mass units to mol, surface to m^2^ and time from minutes to seconds:

(4) $$W=(2005.39)\frac{\left(V)(P\right)}{\left(T_{a}K)(A\right)}$$

Once *e_i_*, *e_o_* and *W* were estimated, *E* can be calculated as established in (5) and expressed in mg/m^2^/s:

(5) $$E=(W)(1000)(18.02)\frac{\left(e_{o}-e_{i}\right)}{\left(P-e_{o}\right)}$$

### 2.3. Stomatal Conductance

Stomatal conductance is another important transpiration dynamic variable that represents the guard cells vapor conductivity [1,2,5]. It can be estimated from primary temperature and *RH* sensors data. The first step is to calculate the leaf saturation vapor pressure *e_leaf_* as a function of *T_leaf_*. For this purpose, (1) can be used to calculate *e_leaf_* substituting *T_a_* by *T_leaf_* to obtain (6). *r_b_* is considered a constant of 0.3 m^2^s/mol. Once *e_leaf_* is obtained, stomatal conductance (*C_leaf_*) can be calculated by using (7), expressing the result in mmol/m^2^/s as was previously utilized [19]:

(6) $$e_{\mathrm{leaf}}=6.13753\times 10^{-3}\text{exp}\left(T_{\mathrm{leaf}}\frac{18.564-\frac{T_{\mathrm{leaf}}}{254.4}}{T_{\mathrm{leaf}}+255.57}\right)$$

(7) $$C_{\mathrm{leaf}}=\frac{W}{\left(\frac{e_{\mathrm{leaf}}-e_{o}}{e_{o}-e_{i}}\right)\left(\frac{P-e_{o}}{P}-R_{b}W\right)}(1000)$$

### 2.4. Vapor Pressure Deficit

*VPD* is a variable that represents the margin between air vapor pressure and air saturation vapor pressure. If air *RH* is low, *VPD* has a large margin; but if *RH* is high *VPD* is low and it is easy to get undesirable condensation conditions [6]. To calculate *VPD* it is necessary to subtract *e_i_* from *e_s_*. The most common units to represent *VPD* are kPa [2]:

(8) $$\mathrm{VPD}=e_{s}-e_{i}$$

## 3. Smart Sensor Methodology

**Figure 2.**
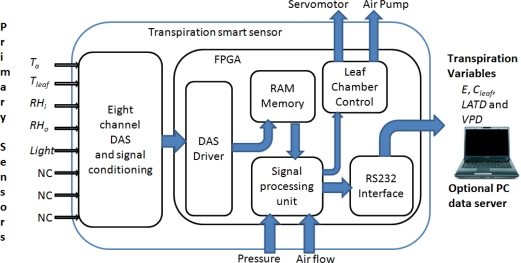
Transpiration smart sensor architecture.

The proposed methodology for the smart sensor can be seen in Figure 2. It consists of the following stages: primary sensors, data acquisition system (DAS), FPGA-based digital signal processing (DSP), RAM memory to storage sensors measurements, RS232 data communication module, and leaf chamber mechanism control system. In the first stage, five primary sensor signals are obtained from two RTD temperature sensors, two *RH* capacitive sensors and one light quantum sensor. The second stage consists of an eight channel DAS capable of acquiring the signals of the five primary sensors and leaving the last three channels disconnected (NC). The signal processing stage is carried out on a FPGA-based hardware signal processing (HSP) unit, as reported by [23] for CNC and [24] for robotics vibration applications. Atmospheric pressure is provided by external smart sensor input. Volumetric air flow is fixed at a constant 0.9 lpm flow rate by using a passive flow limiter. Data communication is carried out via RS232 interface embedded in the FPGA unit to send the measurement to a data server PC or another system. Finally, the leaf chamber opening mechanism and vacuum pump is controlled by the FPGA HSP unit.

### 3.1. Transpiration Smart Sensing Cycle Methodology

**Figure 3.**
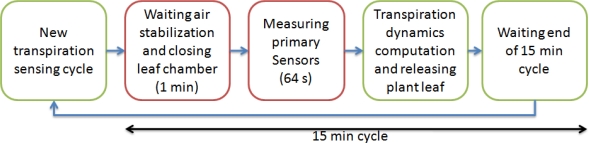
Block diagram of transpiration smart sensing cycle.

Transpiration smart sensing cycle methodology is shown in Figure 3. Here, the green blocks represent the open leaf chamber stages and red blocks represent the closed leaf chamber; therefore, isolating the plant leaf. Each measurement starts with the activation of the air vacuum pump and closing the leaf chamber by the servomotor controller to isolate the plant sample. The smart sensor performs a 1 min delay to wait pneumatic line flow steady state. After that, the data acquisition starts by measuring cycles of 1 Hz sampling frequency from the five primary sensors. This process is repeated to acquire 64 samples from each sensor in each transpiration measurement cycle. Once sufficient data has been acquired, the computation of transpiration dynamics are performed, data can be transferred, the leaf chamber is opened and vacuum pump is turned off to save energy. Finally, another delay is carried out to complete the 15 min duration of the entire transpiration smart sensing process. This measurement period was selected because in commercial equipment, the fastest acquisition period is 15 min and this is necessary to establish the same sampling frequency to compare both sensing techniques.

### 3.2. Transpiration Methodology

**Figure 4.**
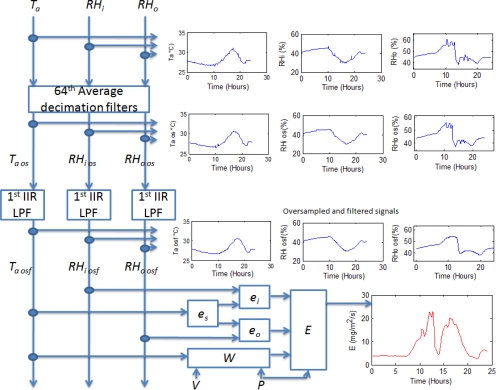
Transpiration estimation signal processing methodology.

In order to calculate plant transpiration *E*, an FPGA-based signal processing methodology is proposed and described in Figure 4. Average decimation filters of 64th order are applied to all the primary sensors signals *T_a_*, *RH_i_*, and *RH_o_* in order to reduce undesired quantization noise as stated in (9), (10), and (11) and presented by [14]. Once *T_a os_*, *RH_i os_*, and *RH_o os_* were estimated, a 1st order IIR low-pass filter (LPF) with cut-off frequency (*f_c_*) of 1/3600 Hz is applied to obtain the filtered versions of the decimated signals known as *T_a osf_*, *RH_i osf_*, and *RH_o osf_*. To gather *E*, equations (1) to (5) are computed from the filtered signals as presented in Figure 4.

(9) $$T_{\mathrm{a os}}\left(\frac{k}{64}\right)=\frac{1}{64}\sum_{i=0}^{64-1}T_{a}(k-i)$$

(10) $${\mathrm{RH}}_{\mathrm{i os}}\left(\frac{k}{64}\right)=\frac{1}{64}\sum_{i=0}^{64-1}{\mathrm{RH}}_{i}(k-i)$$

(11) $${\mathrm{RH}}_{\mathrm{o os}}\left(\frac{k}{64}\right)=\frac{1}{64}\sum_{i=0}^{64-1}{\mathrm{RH}}_{o}(k-i)$$

### 3.3. Stomatal Conductance Estimation Methodology

**Figure 5.**
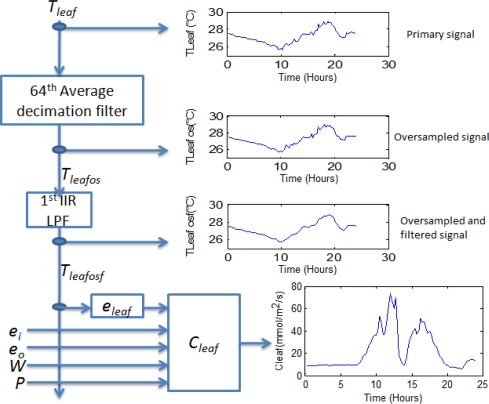
Stomatal conductance estimation signal processing methodology.

Stomatal conductance is advantageous because it utilizes certain factors previously calculated in the transpiration stage. A FPGA-based signal processing methodology is proposed and described in Figure 5.

Average decimation filters of 64^th^ order are applied to *T_leaf_* sensor signals to reduce undesired quantization noise in the same manner as the previous stage according to (12). Once *T_leaf os_* was estimated, a 1st order IIR low-pass filter (LPF) stage with *f_c_* = 1/3600 Hz is applied to obtain its filtered version *T_leaf osf_*. Consequently, *e_leaf_* is calculated as stated in (6) and introduced in (7) to obtain *C_leaf_* :

(12) $$T_{\mathrm{leaf os}}\left(\frac{k}{64}\right)=\frac{1}{64}\sum_{i=0}^{64-1}T_{\mathrm{leaf}}(k-i)$$

### 3.4. Vapor Pressure Deficit Methodology

**Figure 6.**
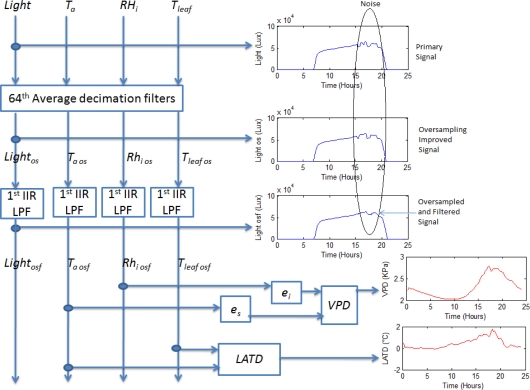
*VPD*, *LATD* and *Light* estimation signal processing methodology.

*VPD* estimation is very simple once *e_s_* and *e_i_* are calculated in the transpiration calculation stage. *VPD* is obtained from the subtraction stated in (13). *VPD* implementation can be noted in Figure 6:

(13) $$\mathrm{VPD}=(100)(e_{s}-e_{i})$$

### 3.5. Leaf-Air Temperature Difference Methodology

*LATD* calculation requires subtracting the filtered air temperature and filtered leaf temperature. As occurred in previous calculation stages, once *T_a osf_* and *T_leaf osf_* was calculated, *LATD* computation is simple by using (14), as demonstrated in Figure 6:

(14) $$\mathrm{LATD}=T_{\mathrm{a osf}}-T_{\mathrm{leaf osf}}$$

### 3.6. Ambient Light Smart Sensor Methodology

To measure ambient light in a smoother signal manner, the same average decimation filter (15) plus 1st order IIR LPF with *f_c_* = 1/3600 Hz was applied to the signal *light* in order to obtain its improved version *light_osf_*:

(15) $${\mathrm{light}}_{\mathrm{os}}\left(\frac{k}{64}\right)=\frac{1}{64}\sum_{i=0}^{64-1}\mathrm{light}(k-i)$$

## 4. Experimental Setup

### 4.1. Experiment Setup

**Figure 7.**
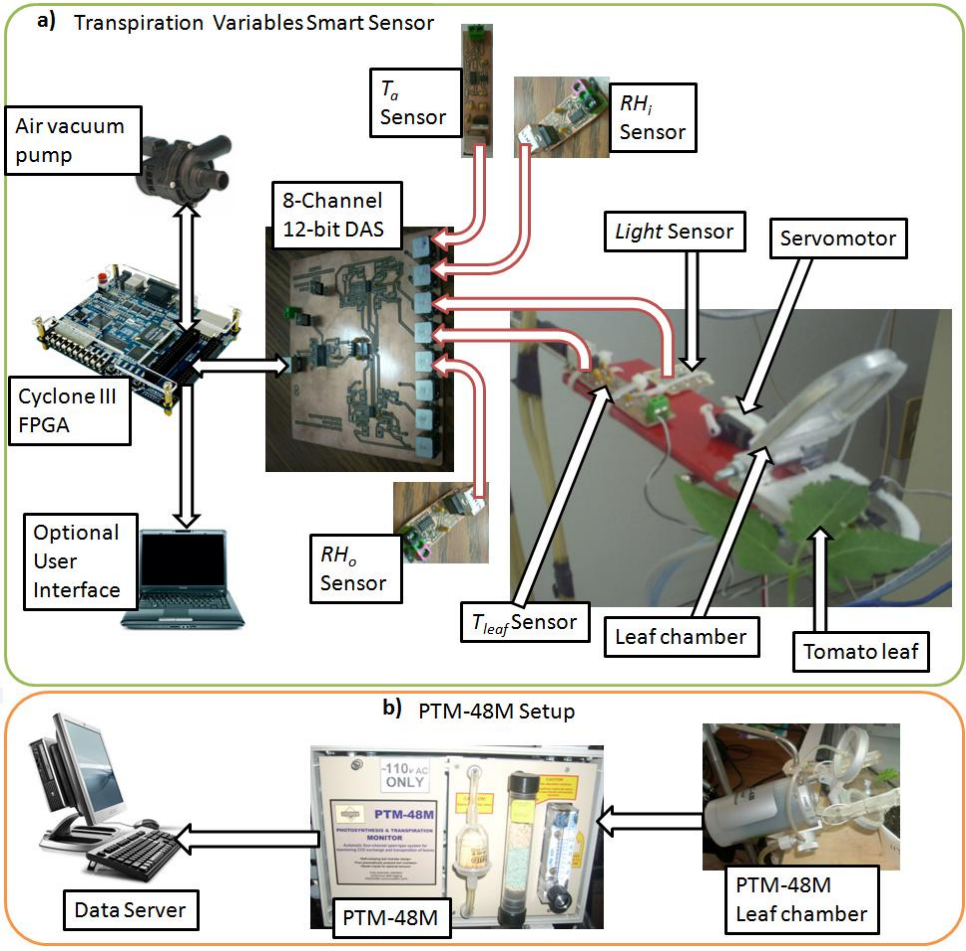
(a) Transpiration smart sensor experimental setup. (b) Phytech PTM-48M setup.

The experimental setup can be seen in Figure 7, which shows the development of a smart sensor setup and the commercial Phytech PTM-48M, see Figure 7a and Figure 7b used for performance comparisons. For this experiment, tomato (*Lycopersicon esculentum*) plants were chosen as biological material to measure transpiration responses in the proposed smart sensor testing. The proposed smart sensor consists on an instrumentation platform capable of measuring *T_a_, T_leaf_, RH_i_, RH_o_* and *Light*. To measure temperature readings, Honeywell Pt1000 RTD primary sensors were used which have a measurement range from −200 °C to 540 °C, but are configured for a 0 to 65 °C range with an accuracy rate of ±0.3 °C considered suitable for plant temperature ranges [10]. A RTD-signal conditioning system was developed to convert the resistance variation into a 0 to 5 volt format. For *RH* measurements, Honeywell HIH-4000 capacitive *RH* sensors with a range from 0 to 95% *RH* and accuracy of ±2.5% were selected and connected through a developed *RH*-signal conditioning system [11]. Ambient light measurement is achieved by using an OSRAM SFH-5711 light sensor with range from 0 to 100,000 lux and an accuracy of ±0.04% of its measured value, providing a 0 to 50 μA current signal [25], converted into 0 to 5 V by a designed light-signal conditioning system. The results are suitable in order to measure light intensities in rooms from total darkness to complete sunlight. Each primary sensor reading passes through a 2nd order analog anti-alias LPF with 20 Hz cut-off frequency embedded in the proposed smart sensor. An eight channel 12-bit data acquisition system based on the Burr Brown ADS7844 analog to digital converter instrumentation platform was developed to read the primary sensors [26]. An FPGA based hardware digital signal processing unit was utilized to compute the transpiration response variables from the primary sensors readings based on an Altera Cyclone III EP3C16F484C6N device with 16,000 LE [27]. For the open/close leaf chamber mechanism, a miniature servomotor model E-Sky 000155 was utilized because of its low power consumption. The air flow was induced using a dc-motor piston based vacuum pump. The FPGA IP core was implemented in VHDL language, integrating the DAS driver, leaf chamber motors control, signal processing unit and communications blocks.

The experiment was designed to monitor transpiration variables every 15 min to compare the proposed smart sensor with a Phytech PTM-48M photosynthesis and transpiration monitor configured at its fastest sampling period which is 15 min [18]. Both were connected to the same tomato plant to prove the effectiveness of the proposed smart sensor. The experiment ran for 24 hours beginning at 12:00AM and finishing at 12:00AM of the next day. It permits the acquisition of four measurement cycles per hour for a total of 96 measurements every 24 h. As was aforementioned, each measurement cycle acquires 64 samples from each primary sensor at a sampling frequency of 1 Hz to apply the 64th averaging decimation filters. The data can be sent to a data server for massive storage via an Analog Devices ADM3232 RS232 transceiver [28].

### 4.2. Primary Sensor Signal Improvement Results

**Figure 8.**
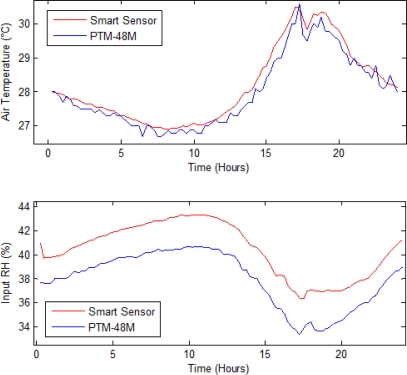
Primary sensors signal comparison. The proposed smart sensor signals are in red and Phytech PTM-48M readings in blue.

In this subsection, a comparison between the *T_a_* and *RH_i_* readings from the proposed smart sensor and the commercial PTM-48M is presented. Figure 8 illustrates this comparison where blue signals correspond to PTM-48M readings and red ones to the proposed smart sensor primary readings. Here, it can be noted that a similar tendency occurs for both measurements, but a lower amount of noise in the red signals is present due to the 64-sample average decimation filters and 1st order IIR filters that reduces undesirable variations. In this manner, the filtering advantages of the smart-sensor signal processing can be clearly noticed. *RH_o_, T_leaf_,* and *Light* readings are not compared because PTM-48M does not provide *RH_o_* measurement in the data output table and does not have *T_leaf_* and *Light* sensors.

### 4.3. Transpiration Results and Comparison

**Figure 9.**
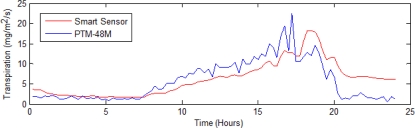
Comparison between the developed smart sensor and the PTM-48M transpiration estimations.

This subsection shows a comparison between the proposed smart sensor transpiration estimation and the reference PTM-48M. In Figure 9, this comparison is represented by using blue for the PTM-48M transpiration signal and red for the smart-sensor transpiration estimation signal. Here, it is noteworthy that a similar transpiration signal behavior between both instruments occurs.

### 4.4. Fused Transpiration Dynamics Smart Sensing Results

**Figure 10.**
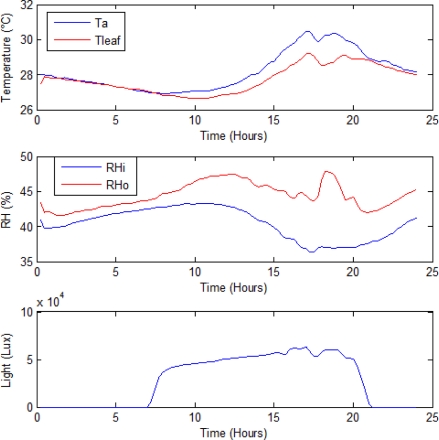
Primary sensor readings of the proposed smart sensor.

The proposed smart sensor fuses *T_a_, T_leaf_, RH_i_, Rh_o_* and *Light* measurements in a single device which is considered highly desirable for precision agriculture applications to be able to monitor different environmental factors that can affect the crops. In Figure 10, the monitoring results of the primary sensors in this experiment are presented. All of these were previously oversampled 64 times for the average filtering process and passed through an IIR 1st order LPF with a cut-off frequency of *f_c_* = 1/(3600). As it can be seen, the amount of noise in these primary signals is very low.

**Figure 11.**
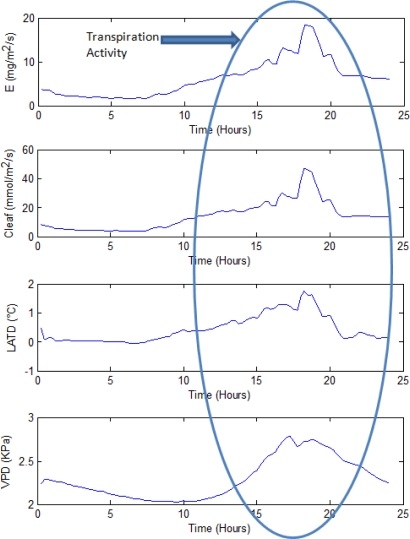
Transpiration dynamics results.

In Figure 11, transpiration dynamics response variables (*E*, *C_leaf_*, *LATD*, and *VPD*) can be observed. These parameters share a similar dynamic behavior because they are related to the entire photosynthesis and transpiration processes which involve different phenomena.

The computation of the required equations to obtain these results is achieved using FPGA reconfigurability and open architecture that permits implementation of any digital system such as data acquisition, memory management, signal processing, and data communication. The developed smart sensor fuses five primary sensors and can measure the necessary environmental variables to calculate transpiration dynamics and permits the user to observe and record different primary and response measurements at the same time and the relationship between these. This is very useful in precision agriculture in order to detect abnormal conditions. In contrast, commercial equipment as noted in [18,19] can measure time series of transpiration information; however, they do not provide the readings for all the primary sensors. In some cases, they are not equipped with the necessary sensors. The FPGA-based unit permits improvements of the primary sensor signals by oversampling and digital filtering that is consequently reflected in superior accuracy, and overall transpiration variables signal quality. The integration of these elements merge different variables at the same time that can be acquired and used to take specific control actions by communicating these transpiration measured values to other systems via RS232 interface like PC data servers, an irrigation controller, or a climatic control unit due to the online processing and remote communications capabilities of the proposed smart sensor. Taken together they constitute a smart sensor solution to monitor transpiration variables in precision agriculture applications by using a single FPGA-based system.

## 5. Conclusions

In this investigation the development of a novel smart sensor that can estimate plant-transpiration dynamic variables as: transpiration, stomatal conductance, leaf-air temperature difference, and water vapor deficit in real time is presented. This smart sensor fuses five primary sensors: two temperature sensors, two relative humidity sensors and one light sensor. To show the effectiveness of the proposed smart sensor, it was compared with a commercial Phytech PTM-48M transpiration monitoring system. Results show that the proposed sensor can obtain very similar results compared to the reference system with less noise due to the digital filtering applied to the primary measurements. The transpiration dynamics variables are calculated in real time from the primary sensor data providing very useful information related to the plant transpiration which is valuable to schedule irrigation, prevent diseases, and detect drought conditions in precision agriculture. Similar behavior of the estimated transpiration variables shows the relationship between these and how they depend on the primary sensor readings. The necessary computations in order to obtain the transpiration dynamics are computed in a low-cost FPGA platform in which parallel architecture is utilized to implement the transpiration equations. This permits the integration of the data communication, memory management data acquisition and signal processing in a single embedded sensor which can be used to monitor plant transpiration variables and their relationships in a wide range of precision agriculture applications. Finally, transpiration related stress conditions can be detected in real time because of the online processing and communications capabilities. All of which constitutes a very useful precision agriculture smart sensor.
